# The emerging role of neutrophil extracellular traps in endometritis

**DOI:** 10.3389/fimmu.2023.1153851

**Published:** 2023-03-22

**Authors:** Hongyan Li, Ling Liu, Junrong Wang, Weiliang Zhao

**Affiliations:** ^1^ Department of Urology, China-Japan Union Hospital of Jilin University, Changchun, China; ^2^ Department of Pediatrics, China-Japan Union Hospital of Jilin University, Changchun, Jilin, China; ^3^ Department of Obstetrics and Gynecology, China-Japan Union Hospital of Jilin University, Changchun, Jilin, China; ^4^ Department of Anesthesiology, China-Japan Union Hospital of Jilin University, Changchun, Jilin, China

**Keywords:** endometritis, NETs, injury, selenium, bacteria

## Abstract

Endometritis is a kind of common obstetric disease in women, usually caused by various pathogenic bacteria. Neutrophil infiltration is one of the most important pathological features of endometritis. Neutrophils can reach the uterine cavity through the endometrium, and make early response to the infection caused by the pathogen. Neutrophil extracellular traps (NETs), a meshwork of chromatin fibers extruded by neutrophils, have a role in entrapping microbial pathogens. It has been confirmed that NETs have a strong antibacterial effect and play crucial roles in the occurrence and development of various diseases. However, while killing pathogenic bacteria, excessive NETs formation may cause immune damage to the body. NETs are present in endometrium of female domestic animals in different physiological periods, especially post-mating, postpartum and in the presence of lesions, especially in endometritis. Meanwhile, NETs and its products might contribute to a reduction in physical clearance and persistent endometritis. In brief, NETs is a double-edged sword and it may play a different role in the development of endometritis, which may be beneficial or harmful, and its specific mechanism needs further study. Here we provide an overview of the role of NETs in the development of endometritis and the regulatory role of selenium on NETs formation and endometritis.

## Introduction

Endometritis is a kind of obstetric disease, which often occurs in women and animals. It can cause infertility and reduce the reproductive performance (1). The pathogenic factors of endometritis are complex, mainly caused by bacteria, such as *Staphylococcus*, *Escherichia coli*, *Streptococcus*, etc. (2). According to clinical manifestations, endometritis can be divided into chronic and acute types. As the outer surface of the uterine cavity, endometrial epithelial cells first contact with the pathogenic bacteria, which are an important part of the innate immunity of the uterus and play a sentinel role in the innate immune defense. Therefore, when the integrity of endometrial epithelial cells is destroyed, it will be more conducive to the invasion and infection of pathogenic microorganisms.

Once the endometrium is attacked by pathogenic microorganisms, the innate immune system performs its initial defense function. With the invasion of pathogenic bacteria into the uterus to cause an inflammatory response, neutrophils can reach the uterine cavity through the endometrium, and make early response to the infection caused by the pathogens (3). Mounting evidences suggest that the number of neutrophils increases in the late pregnancy of healthy dairy cows, performing a strong phagocytic ability. However, when endometritis and other uterine diseases occur, the numbers and phagocytic ability of neutrophils are reduced. The numbers and phagocytic ability of neutrophils are lower in the postpartum compared with the prenatal (4). Because the phagocytic capacity of neutrophils in uterine cavity is consistent with that in peripheral blood, the function of neutrophils in uterine cavity is often evaluated by peripheral blood neutrophils.

Neutrophils, as an integral part of innate defense system, play an important role in the body’s defense against pathogen invasion. Neutrophils mainly kill pathogens through phagocytosis and degradation (5). In 2004, Brinkmann et al. first discovered that neutrophils can kill pathogenic microorganisms by releasing a network structure (6). This structure is named NETs, which are different from phagocytosis and degradation, and are a new way of immune response. NETs play crucial roles in the occurrence and development of various diseases. However, there are few reports about the role of NETs in endometrium, especially in the pathogenesis of endometritis. In this review, we will focus on the role of NETs primarily in endometritis. In addition, we provide some latest information on NETs formation and mechanism of action in the course of many disorders.

## NETs formation

NETs were formatted by which activated neutrophils release their DNA and intracellular components in a network structure. Several proteins adhere to NETs, including histones and granular content, among which are components with bactericidal activity. NETs formation occurs as specific proteases are translocated into the neutrophil nucleus, which causes chromatin decondensation through citrullination (7). At present, it has been proved that NETs can be induced by many substances. In addition to pathogenic microorganisms, LPS, MPO, activated platelets, TNF-α and PMA can stimulate neutrophils to produce NETs (8). However, due to the differences in signal pathways of different stimuli and the different stages of NETs formation, the mechanisms involved are not exactly the same. Although the exact molecular mechanism of the NETs formation is not clear, more and more studies show that the formation and regulation of NETs depend on the production of ROS mediated by NADPH oxidase. ROS has a prominent role in NETs formation. Some scholars believe that the production of ROS is a prerequisite for NETs formation, which can induce the activation of protein kinase C (PKC) (9), the activation of Raf/MEK/ERK signaling pathway and the increase of intracellular calcium ions concentration. There are a large number of NADPH oxidase on the cell membrane and phagosome membrane of neutrophils. When stimulated by signal, ROS produced is present in the cell or released to the outside of the cell, playing a role in killing pathogens (10, 11). In patients with chronic granuloma, ROS production is inhibited by mutations of CYBB gene encoded by NADPH oxidase 2(NOX2), and polymorphonuclear neutrophil (PMNs) cannot form NETs (12). Inhibition of NADPH oxidase activity by diphenyliodonium (DPI) can affect ROS production and effectively block PMA induced NETs formation (13). In addition, some enzymes play an important role in NETs formation, such as neutrophil elastase (NE), MPO and peptidylarginine deiminase type 4 (PAD4). NE and MPO are released from neutrophils azurophilic granule and transposed into the nucleus to participate in the degradation of histones and the loosening of chromatin (14). Ribozyme PAD4 promotes the degradation of histones by neutrophils elastase, and changes its amino acid structure to convert arginine to citrullinic acid, resulting in the loss of positive charge necessary for histone to interact with DNA, leading to the generation of chromatin depolymerization and inhibition of NETs formation (15). It has been found that the use of PAD4 inhibitor in SLE mice models can reduce the formation of NETs and prevent lesions of skin, kidney and blood vessels (16).

## Antimicrobial activity of NETs

NETs are based on DNA and are accompanied by a variety of protein particles, including MPO, histones, elastin, and lysozyme (17). NETs mainly rely on its unique three-dimensional network structure to capture pathogens. After capturing the pathogens, NETs can kill the pathogens through its own various antibacterial proteins, and that help other immune cells to function (18). The mechanism by which NETs capture pathogens is yet unclear. In the proposed mechanism, the main idea is the charge attraction formed between positively charged NETs components and negatively charged pathogen components. Currently, *in vivo* and *in vitro* experiments have confirmed that NETs can kill Gram-positive bacteria, Gram-negative bacteria, T. berghei, fungi, parasites, and even capture human immunodeficiency virus (HIV) (19). Meanwhile, based on the dual roles of neutrophils in host immune system, recent research has revealed the two roles played by NETs: as a first line of defense against microorganisms and as a contributor to the pathogenesis of various illnesses (20).

## Immune injury of excessive NETs formation

In the more than 10 years since NETs were discovered, it has been confirmed by a large number of experiments that NETs have a strong antibacterial effect and play crucial roles in the occurrence and development of various diseases. However, while killing pathogenic bacteria, the immune damage to the body itself should also be taken seriously. For example, it was reported that there are NETs in peripheral blood neutrophils of sepsis. NETs can catch and kill pathogens during local infection. However, acute lung injury caused by toxicity to alveolar epithelial cells and disseminated intravascular coagulation (DIC) induced by effects on endothelial cells and coagulation system in systemic infection further increase the mortality of sepsis (21). Saffarzadeh et al. demonstrated *in vitro* studies that NETs can cause harm to alveolar epithelial cells and endothelial cells. When they kill pathogens, neutrophils may release too much NETs, which will affect the body as the degradation of NETs is not timely. Fuchs et al. found that NETs provide scaffolds for the formation of blood clots and stimulate their formation due to their unique network structure (22). The formation of NETs in blood vessels can make platelets adhere, aggregate and activate. Meantime, Vonbrush et al. reported that NETs can promote deep vein thrombosis by binding and activating coagulation factor XII (23). It’s reported that large amounts of NETs will accumulate in the hepatic sinusoids in the systemic inflammation of mice induced by LPS injection, thereby causing blockage and damage to liver cells. NETs are also closely related to autoimmune diseases. For example, large amounts of NETs produce in patients with systemic lupus erythematosus, and their degradation process is inhibited by the combination of NETs and autoantibodies. The presence of large amounts of DNA and histones will induce the body to produce corresponding antibodies, causing immune damage to the host (24). Studies have also been reported that NETs are closely related to the pathological processes of preeclampsia, sepsis, coronary heart disease, and Ferti syndrome.

## NETs and infectious diseases

Bacterial and fungal pathogens can stimulate NETs formation. Studies showed that *Staphylococcus aureus*, *E.coli*, and *Salmonella typhimurium* can be effectively trapped within NETs and eliminated by components of NETs *in vitro* (25, 26). According to their research, NETs may use the DNA-associated elastase to degrade their virulence factors (6). NETs are able to capture, immobilize, and eliminate *Escherichia coli*. Nevertheless, it indicates that NETs were observed in bacteria-induced intestinal diseases and contributed to the injury of intestinal epithelium (27).

Virus induction of NETs formation is now well established. Influenza A-stimulated NETs are dependent on PAD4 (28). NETs may trap and eliminate HIV through myeloperoxidase and α-defensins (29). NETs formation prevents respiratory syncytial virus (RSV) dissemination (30). A recent study shows that NETs effectively control acute Chikungunya virus infection (31).

NETs can be found in the circulation or infected site of patients infected with fungi or parasites. Neutrophils has the ability to trap and eliminate *Candida albicans* through releasing NETs (32). Neutrophils can limit the dissemination of *Toxoplasma gondii* by trapping and killing it *via* NETs release, subsequently showing that NETs formation is MEK-ERK dependent (33). Pathogenic microbes invasion promotes NETs formation to immobilize pathogens and hinder their spread. Meanwhile, different kinds of pathogenic microbes will form various immune escape mechanisms in the process of evolution to evade the host immune system. *Staphylococcus aureus* secretes several virulence factors including leukotoxin GH (LukGH) and Panton–Valentine leucocidin (PVL) to promote NETs through an oxidative pathway-independent mechanism (34, 35). Similar research showed that NETs in the cerebrospinal fluid (CSF) of patients with pneumococcal meningitis promote pneumococcal survival and reduce bacterial clearance (36). *Cryptococcus neoformans* possesses a capsular polysaccharide glucuronoxylomannan(GXM) that improves virulence by mediating resistance to NETs (37).

## NETs and endometritis

### NETs formation in endometritis

The formation of NETs has been observed in the development of endometritis (38). As we mentioned earlier, many kinds of bacteria, fungi, viruses and parasites can induce NETs formation, and many of them can cause endometritis in cows. After endometritis in cows, neutrophils infiltrate through the endometrium and enter the uterine cavity. Meanwhile, due to the presence of pathogenic bacteria, endotoxins and increased oxygen free radicals, these factors are sufficient conditions for the formation of NETs (39).

In addition to the above, the immunometabolic requirements for NET release are still ambiguous, including requirement for some macro- and micronutrients. It has been reported that NETs formation is affected by some trace elements such as zinc, iron and copper (40). And calcium ion plays an important role in a series of signaling cascade processes that NETs formation depends on (41). In a study on some of the metabolic requirements for NET formation, Rodriguez-Espinosa et al. suggested that NET formation contained two phases. It is necessary to study the relationship between postpartum energy metabolism disorder and endometritis and NETs formation.

### Positive effect of NETs on endometritis

NETs is closely related to human pregnancy diseases (42). Similarly, NETs formation has been related to endometriosis of domestic animals in the mare with endometritis, and in the post-partum cow (39, 43). Some studies have shown that all bacteria found to cause endometritis in mares may be trapped in NETs. But it seems that different bacteria can induced NETs with the different potency (39). The ability of horse neutrophils to damage different bacteria may be the mechanism of antagonizing some microbes that cause endometritis in mares. Thus, NETs formation might be the mechanism against pathogens responsible for equine endometritis (44).

According to the study of the mare endometritis, the stimulation of semen deposition induced a mass of PMNs invasion which eventually led to post-mating inflammatory responses of the uterus. With the aid of the NETs formation subsequently, sperm would be phagocytosed (45). In addition, activated PMNs would tangle and kill microbes by extruding nuclear DNA and histones to form NETs (46). These reactions can ensure the elimination of sperm and bacteria and then the recovery of endometrium, prepared for conception.

### Negative effect of NETs on endometritis

Interestingly, NETs may have dual actions on the mare endometrium. While PMNs have favorable effect on infection issues as first line in immune defence, they may also release molecules that might damage surroundings inflame issues (47). Previous studies have confirmed that NETs and its related histones may cause damage to epithelial cells and endothelial cells, leading to lung, liver and kidney damage (44). Other findings demonstrated NETs and its component histone could cause injury to bovine mammary epithelial cell (BMEC) *in vitro (* 48). We have reason to suspect that NETs and its components may also have some inevitable connection with endometrial epithelial damage through some ways. In recent study *in vitro*, although not tested *in vivo*, elastase, cathepsin-G and myeloperoxidase which were released by NETs may result in impaired prostaglandin E2 (PGE2) release in the mare endometrium. This might contribute to a reduction in physical clearance and persistent endometritis.

Despite many breakthroughs in the study of the mechanism and key links of NETs formation, will there be a large number of NETs formation *in utero*? What role do NETs play in endometrial infection? Do NETs affect the expression of key junction proteins, such as apoptosis, pyrolytic associated inflammatory bodies and caspase signaling pathway? All these problems need to be clarified by research.

### Effects of selenium on NETs formation and endometritis

Selenium (Se) is a non-metallic trace element that has important effects on human and animal bodies. It has been reported to exhibit anti-tumor, anti-heavy metal, anti-virus, anti-oxidation, and enhancing immunity effects (49, 50). Recent studies demonstrated selenium could inhibit LPS-induced endometritis in mice (51). Meanwhile, selenium had protective effects in *S.aureus*-induced endometritis in rats (52). Also, selenium deficiency could aggravate inflammatory response in the mice uterus (53). A large number of studies demonstrated that selenium could inhibit the formation of NETs. Selenium could suppress Fumonisin B1-induced NETs formation in chicken neutrophils (54). In addition, selenium could inhibit lead (Pb)-induced NETs formation (55). A previous study demonstrated that selenium induced NETs formation in the progression of arteritis and silencing-SelS worsened arteritis (56). These reports indicated selenium may inhibit NETs formation through NETs formation. And NETs can be used as a target for the treatment of endometritis.

## Conclusions

It has been confirmed that NETs have a strong antibacterial effect and play crucial roles in the occurrence and development of various diseases. However, while killing pathogenic bacteria, excessive NETs formation may cause immune damage to the body. NETs are present in endometrium of female domestic animals in different physiological periods, especially post-mating, postpartum and in the presence of lesions, especially in endometritis, playing a key role of immune clearance, protection and regulation. Meanwhile, NETs and its products might contribute to a reduction in physical clearance and persistent endometritis. In conclusion, NETs may play different roles in endometritis, which may be beneficial or harmful, and its specific mechanism needs further study. It may be the key target of prevention and treatment of endometritis and other infectious diseases in dairy cattle to regulate the immune system and maintain the appropriate levels of NETs in blood and local areas.

## Author contributions

HL, LL, JW and WZ wrote the manuscript. WZ revised the review. All authors contributed to the article and approved the submitted version.
